# Articulation and Dynamics Influence the Perceptual Attack Time of Saxophone Sounds

**DOI:** 10.3389/fpsyg.2018.01692

**Published:** 2018-09-24

**Authors:** Toni Amadeus Bechtold, Olivier Senn

**Affiliations:** Department of Music, Lucerne University of Applied Sciences and Arts, Lucerne, Switzerland

**Keywords:** perceptual attack time, synchronicity, music, rhythm, timing, saxophone

## Abstract

Perceptual attack time (PAT) is defined as the moment when the most salient rhythmical feature of a sound is perceived. This paper focuses on the PAT of saxophone sounds, investigating how the location of this point in time changes when a note is played with different characteristics. Nine saxophone sounds that differ in articulation and dynamics were examined. Ground truth for PAT was determined in a synchronization judgment experiment with 40 participants. Articulation (*p* < 0.001, η^2^ = 0.316), dynamics (*p* < 0.001, η^2^ = 0.098), and their interaction (*p* < 0.001, η^2^ = 0.094) affected the placement of the PAT. The onset rise time, which has been used as a predictor for PAT in earlier studies, was only weakly correlated with PAT (*r* = 0.143, *p* = 0.006).

## Introduction

**Figure 1:**
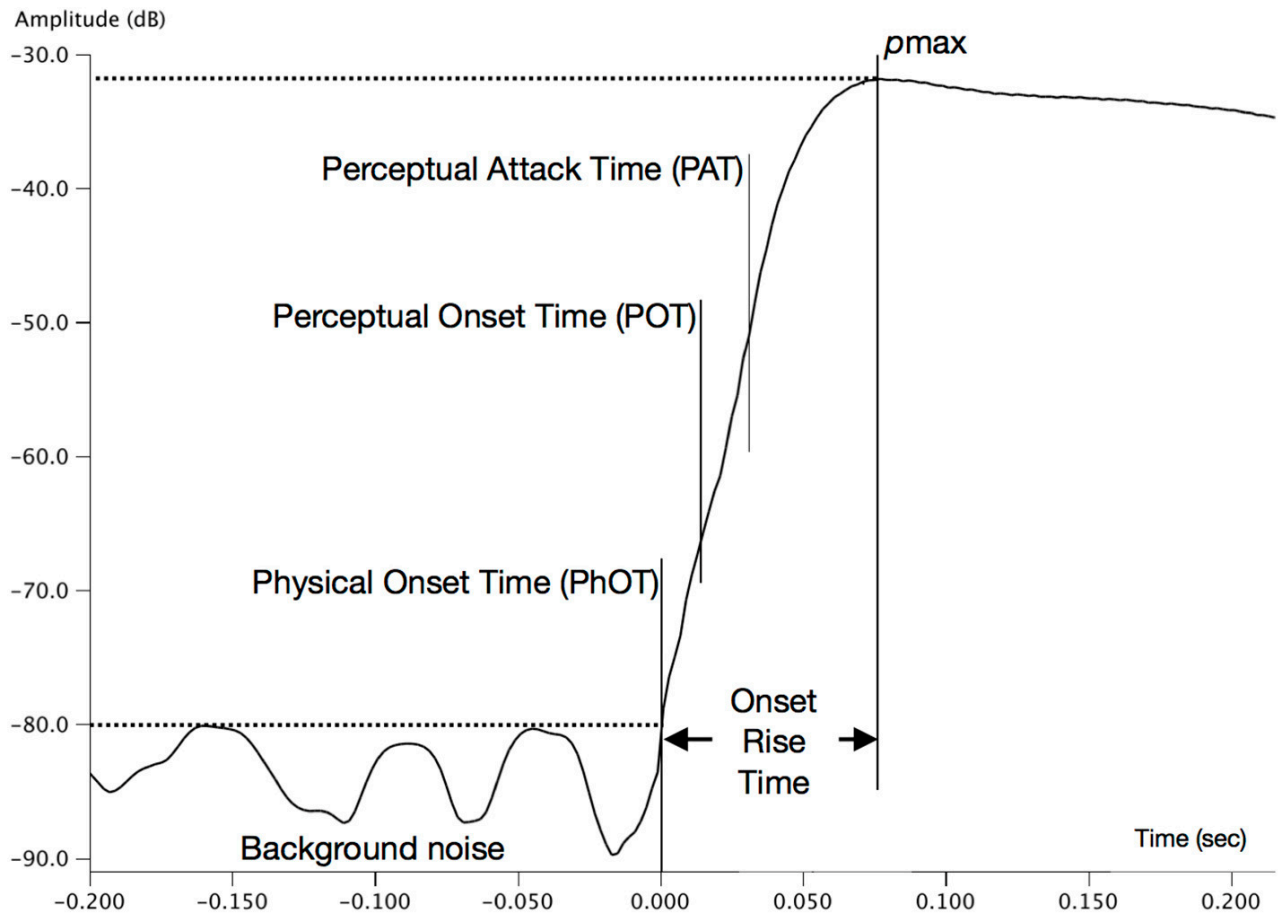
Example intensity plot of the beginning of one of the stimuli used. Important points in time for this study are marked. Physical Onset Time (*PhOT*) and the sound's point of first maximal intensity (*p*max) were estimated by eye. The time between *PhOT* and *p*_max_ is the *onset rise time* of the stimuli. The Perceptual Attack Time (*PAT*) was experimentally determined. Perceptual Onset Time (*POT*) was calculated according to Collins (2006).

The perception of musical sounds in time is the basis for crucial concepts of music cognition like rhythm, meter and groove. But at which point in time exactly do we perceive the beginning of a sound? Acoustically, a sound starts with the *physical onset*, which is the moment when its amplitude exceeds the background noise (Wright, 2008; see also Figure 1). This initiates the sound's attack phase, whose shape has been found to greatly influence the perceptual characteristics of the sound (Elliott, 1975; Hajda, 2007). The exact measurement of *physical onset times* (*PhOT*) plays a central role in music interpretation analysis: the study of microtiming, expressive timing, or tempo rubato discusses the relationships between inter-onset-intervals, which are defined as time intervals between *PhOTs* (Repp, 1992, 1995; Friberg and Sundström, 2002; Belfiglio, 2008; Senn et al., 2009, 2012, 2016; Kilchenmann and Senn, 2011).

Vos and Rasch (1981) introduced the *perceptual onset time* (*POT*), which is the moment when a sound is first perceived. They found that the delay time between the *PhOT* and the *POT* depends on a sound's acoustical properties. This implies that inter-onset intervals based on the measurement of the *PhOT* might prove to be spuriously precise, and to represent rhythm poorly. Gordon (1987) expanded on this idea by introducing the *perceptual attack time* (*PAT*). He defined the *PAT* as the point in time, when the most salient rhythmical feature of a sound is perceived. A more recent definition understands it as the perceived moment of rhythmic placement (Wright, 2008). An equivalent concept in phonetics is the *perceptual center*, or *p-center* (Marcus, 1981; Howell, 1988; Scott, 1998; Barbosa et al., 2005). In theory, inter-onset intervals based on the *PAT* represent humans' perception of sounds in time most accurately. However, *PAT* and *POT* are subjective measures, which rely on the perception of each individual listener (in contrast to the *PhOT*, which is acoustically defined). While *PhOT, POT*, and *PAT* have been found to be (nearly) identical for percussive instruments (Gordon, 1987), other instruments like bowed strings or reeds can produce sounds with *POTs* and *PATs* that are substantially later than their *PhOTs*. In extreme cases, for example in sounds that fade in gradually for several seconds, there might be no *PAT* at all, because there is no salient rhythmic feature.

The attack phase of a sound ends at its maximum intensity (*p*max, see Figure 1). The interval between *PhOT* and *p*max is called *onset rise time. PAT* and *POT* are always within this interval. To use the *PhOT* in timing studies on percussive instruments (like the piano or the drums) is reasonable, because the *PAT* does not differ much from the *PhOT*. This might be one of the reasons why most microtiming studies examined instruments with percussive attack. In non-percussive instruments with a longer *rise time* (Friberg and Sundström, 2002 for saxophone and trumpet, Barthet et al., 2011 for clarinet) timing measurements are likely to be less precise due to the time differences between *PhOT* and *PAT*. A timing analysis based on the *PhOT* potentially misrepresents perceived time relationships (Camp et al., 2011).

Wright (2008) and Polfreman (2013) do not define *PAT* as a single point in time. Rather, for each time point, they estimate the probability of hearing the *PAT* of a sound onset. This results in what they called a *PAT probability density function* or *PAT-pdf*. It builds on Gordon's idea that a time point estimate might be inadequate for representing the *PAT* of a sound with a long *rise time*. This also echoes Rasch's (1988) finding on sound perception that longer *onset rise times* lead to more tolerance for asynchrony in listeners. Asynchronies between bowed string sounds are more acceptable to listeners than similar asynchronies between drum sounds.

Several studies since the 1980s investigated *PAT* localization (Gordon, 1987; Rasch, 1988; Vos et al., 1995; Collins, 2006; Wright, 2008; Villing, 2010; Camp et al., 2011; Polfreman, 2013; Nymoen et al., 2017). Due to its subjective, perceptual nature, the *PAT* cannot be measured on the basis of the sound signal alone. Instead, the *PAT* studies established a perceptual ground truth on *PAT* in a listening experiment first. Subsequently, existing or newly created models for *PAT* were applied to fit the experimental data. These models can be used for automatic PAT detection, but results are not reliable yet (Nymoen et al., 2017).

On which parameters can *PAT* estimation be based? Vos and Rasch (1981) and Gordon (1987) suggested that the duration of the *onset rise time* might be an important factor for the location of the *PAT*. Hence, some of their *PAT* estimation models feature the *onset rise time* as a major predictor. Studies that investigated how we perceive the temporal order of sound events found that sounds with shorter *rise time* were perceived to be earlier than sounds with identical *PhOT* and longer *rise time* (Hirsh, 1959; Barnett-Cowan et al., 2012). Based on these studies, we expect sounds with a shorter *rise time* to have an earlier *PAT* (relative to the *PhOT*), than sounds with a long *rise time*. To our knowledge, the influence of different playing techniques on the *rise time* and on *PAT* has not yet been investigated for wind instruments.

**Figure 2:**
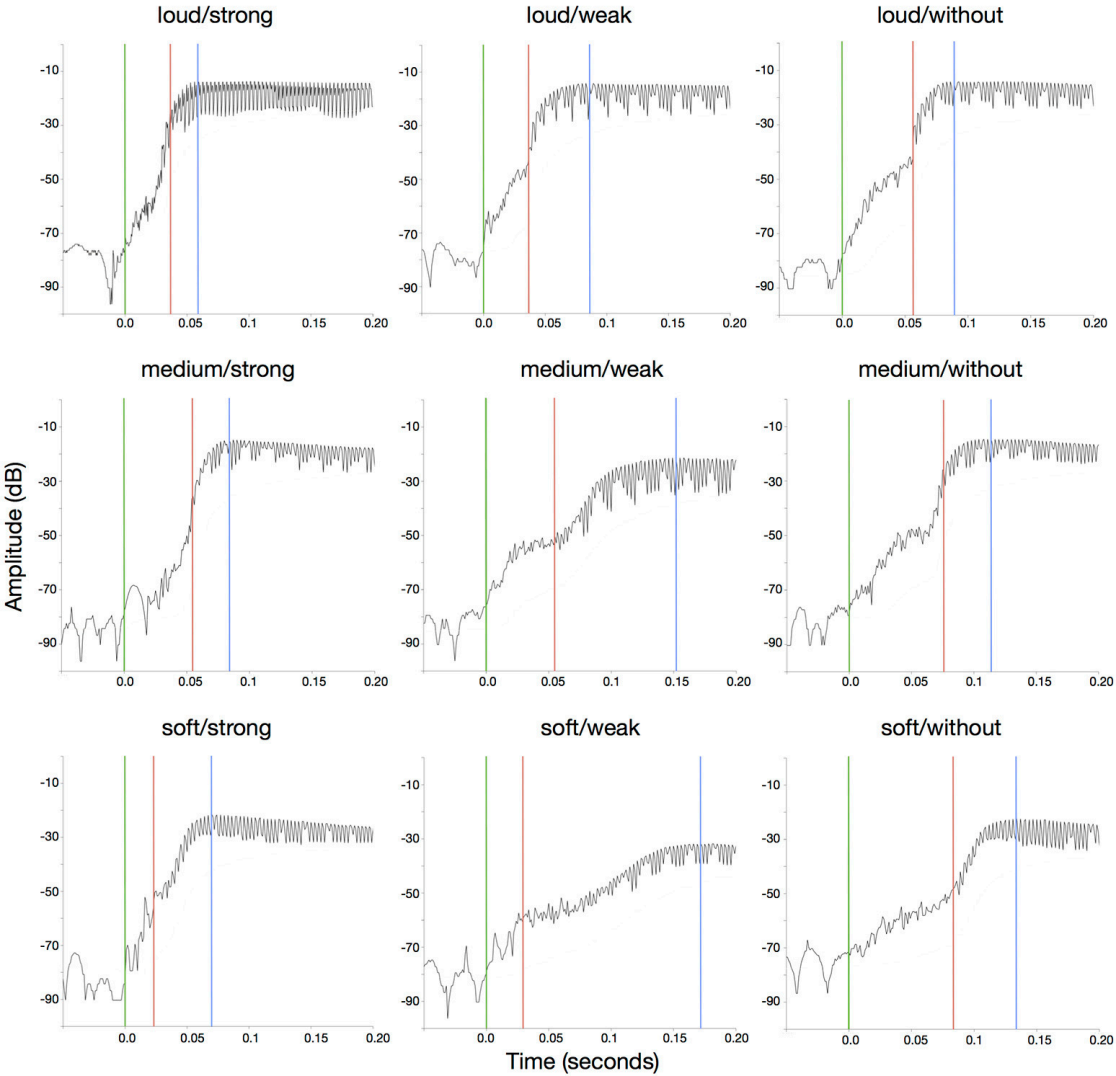
Intensity graphs showing the beginning of the nine saxophone stimuli. Green lines indicate *PhOT*; red lines indicate mean *PAT*; blue lines indicate *p*_max_. The x-axis is time in seconds; the y-axis represents the loudness of the audio signal.

Other *PAT* estimation models investigate the envelope slope or envelope shape as predictors for *PAT*. *PAT* prediction models work well for categories with simple envelopes (as classified by Schutz and Vaisberg, 2014), like *percussive* sounds with decaying envelope and sounds with a *flat* (trapezoidal) envelope. The envelopes of saxophone sounds, however, are complex and diverse (see Figure 2), and they are likely to be influenced by *articulation* and *dynamics*.

Collins (2006) and Villing (2010) compared several *PAT* or *p-center* prediction models. They came to the conclusion that there is no single model that is useful and reliable for all kinds of sounds yet. Collins pointed out that there are indeed some models that successfully predict the *PAT* for a subset of stimuli with similar properties (for example for sinusoidal tones), but fail with respect to another subset with different characteristics. For each model, Villing identified sound characteristics that distorted *PAT* prediction. But neither Collins nor Villing specify which models are most successful in estimating the *PAT* of complex saxophone sounds.

*PAT* prediction models are often based on a large sample of stimuli with different sound characteristics. Sounds were either played on ordinary musical instruments, or they were synthetically created (Collins, 2006; Polfreman, 2013), like sinusoidal tones. The use of a variety of sounds and instruments is a precondition for creating universal prediction models. Yet, this generality comes at the price of reduced detection reliability.

Gordon (1987) discussed the *PAT* of different instruments or instrument families (like reed instruments) separately. He also compared *PATs* for two different types of saxophones (alto and soprano) and found their *PATs* to be different. To our knowledge, no study has focused on one single instrument only.

Additionally, Gordon (1987) tested *dynamics* as a potentially relevant factor in one case: he compared a very soft saxophone sound (*pp*) to a louder one (*mf*), resulting in different *PATs*. This echoes results by Vos and Rasch (1981) who showed that sounds with lower intensity have a later *POT* compared to sounds with higher intensity, but did not examine the effects on the *PAT*. So far, *articulation* (the playing technique used to initiate a sound) has not been studied as a predictor for the PAT. Yet, from a musician's viewpoint, articulation is an essential property of a note. Since articulation happens at or near the beginning of a note, we expect it to be important for the attack phase and thus influence the *PAT*. A sharper *articulation* has a more percussive quality, and more percussive notes are generally associated with shorter *rise times* and earlier *PAT*s.

This study examines *PATs* of tenor saxophone sounds. We aim to test the hypothesis, that the playing technique of a saxophone sound has an influence on its *PAT*. Specifically, we hypothesize that *articulation* (in this study operationalized as the strength of the tongue attack) and *dynamics* (the loudness with which the sounds are played) are associated with the location of the *PAT* in saxophone sounds. We expect loud and strongly articulated sounds to have an earlier *PAT* than soft and weakly articulated sounds. Additionally, the study investigates whether the *onset rise time* is related to the *PAT* and to what extend the *rise time* can be used as a predictor for the *PAT* in saxophone sounds.

## Method

### Stimuli

For this study, nine different saxophone sounds were used as stimuli. The sounds were played by the first author, who is a professional tenor saxophone player, and recorded with a ZOOM H4 Handy Recorder using an external AMT LS Studio Saxophone Microphone in a STUDIOBOX professional sound booth. Distance between the clip-on microphone and saxophone bell was kept constant throughout the recordings. The same saxophone (Conn New Wonder II/Transitional Model), mouthpiece (Otto Link Super Tone Master 7^*^), ligature (François Louis Ultimate) and reed (François Louis #3) were used throughout the recording sessions.

The sounds were varied in terms of *dynamics* and *articulation*, but were played as similar as possible otherwise. Sounds either had soft, medium, or loud *dynamics*. *Articulation* had also three levels: sounds were played either without tongue attack, with weak tongue attack, or strong tongue attack. Both, *articulation* and *dynamics*, affect continuously differentiable physical properties (like air pressure or air flow speed), hence the categorization into three levels per variable is somewhat arbitrary. However, the dynamics levels were chosen to be clearly differentiated and to correspond to common musical categories (*piano, mezzoforte, forte*). In *articulation* without tongue attack the tip of the tongue does not impede the airflow before the release. This is categorially different from the two articulation categories with tongue attack, which are characterized by the tongue interrupting the airflow at the reed before the note is released. The distinction between weak and strong tongue attack is a gradual distinction: the weak attack corresponds to a balanced articulation, whereas the strong attack corresponds to an accentuated note.

All stimuli were played on the same *pitch* that was chosen from the middle register of the instrument (Eb4 concert, ~311 Hz), and is both comfortable to play and agreeable to listen to. The recordings were made in 9 consecutive sessions, one for each sound with the same *dynamics* and *articulation* feature combination. In each session, at least 10 versions of the same sound were recorded. From these versions, the player selected one sound that had a pleasant sound quality, that showed good synchronization of tongue and airflow, and that clearly belonged into the corresponding *dynamics* and *articulation* categories, judged by ear. The differences in *dynamics* for each *articulation* category can be seen in Figure 2.

RMS levels for the sounds were calculated to verify that the selected sounds fit the intended *dynamics* levels (Table 1). Two sounds (medium/weak and soft/weak) are very soft within their dynamic condition. In order to verify whether the notes in the three *dynamics* categories are perceptually well differentiated, we conducted an additional small-scale listening experiment. Participants (*N* = 12, seven participants were professional saxophone players) were presented with the nine stimuli and were asked to rank them from loudest to softest. An analysis of variance showed that there were significant differences between the stimuli's rankings [*F*_(8)_ = 165.2, *p* < 0.001, η^2^ = 0.930]. Follow-up Tukey HSD tests showed that the three stimuli within the loud category were each ranked as louder compared to each of the medium stimuli (*p* < 0.001 for all nine pairwise comparisons), which in turn were ranked as louder than each of the soft stimuli (*p* < 0.001 for all nine pairwise comparisons). Hence, we are confident that our categorization for *dynamics* agrees with perception. The complete data of this experiment and pair-wise comparisons of the stimuli can be found in the Supplementary Materials.

**Table 1:** RMS loudness and Peak levels for the nine stimuli.

**Characteristic**	**RMS loudness (dB)**	**Peak level (dB)**
Loud/strong	−17.1 dB	−7.7 dB
Loud/weak	−18.1 dB	−8.1 dB
Loud/without	−18.7 dB	−8.0 dB
Medium/without	−21.2 dB	−8.6 dB
Medium/strong	−22.0 dB	−8.7 dB
Medium/weak	−25.9 dB	−15.3 dB
Soft/strong	−27.9 dB	−15.5 dB
Soft/without	−29.4 dB	−16.4 dB
Soft/weak	−36.9 dB	−25.7 dB

The *physical onset time* (*PhOT*) of each sound was determined by eye in a signal intensity plot using the *LARA* software (https://www.hslu.ch/en/lucerne-school-of-music/forschung/perfomance/lara/, version 2.6.3). The background noise level of the recordings was determined, and the point of time when the intensity rises above this background noise level threshold for the first time was marked as *PhOT*. This marker was additionally crosschecked in an oscillogram. Automatic *PhOT* detection was not attempted, since at least some sounds may not be percussive enough for automatic detection to work reliably (see Collins, 2005 for a comparison of methods). Thereafter, the maximum intensity at the end of the attack phase of each sound *p*max was located by eye in a signal intensity plot and marked. The *onset rise time* was calculated as the difference between *p*max and *PhOT*.

The *duration* of the selected sounds differed slightly (mean = 0.564 s, *sd* = 0.077 s). Hence, stimuli *duration* is a potential confounding variable in the investigation (Hirsh, 1959; Moore, 2003; Boenke et al., 2009; Barnett-Cowan et al., 2012). However, the correlation between *duration* and the 360 *PAT* estimates was weak [*t*_(358)_ = 2.685, *p* = 0.007, *r* = 0.140], and the correlation between *duration* and *rise time* of the nine stimuli was not significant [*t*_(7)_ = 0.406, *p* = 0.696, *r* = 0.151]. We therefore ruled stimuli *duration* out as a confounder.

Table 2 shows the *onset rise time* of the nine stimuli. Loud *dynamics* and/or strong *articulation* are associated with a low *rise time*.

**Table 2:** Onset rise times of the nine sounds.

**Dynamics**	**Articulation**		
	**Strong tongue attack**	**Weak tongue attack**	**Without tongue attack**
Loud	0.060 s	0.087 s	0.090 s
Medium	0.085 s	0.151 s	0.114 s
Soft	0.070 s	0.173 s	0.134 s

Two sounds with weak tongue attack showed a longer *onset rise time* than those without tongue attack, which is counterintuitive. This observation is most likely linked to the two different methods for building up and releasing the air pressure, which the player uses to set the reed in motion. When, on one hand, the sound is produced without the tongue, the pressure needs to be created and released by an impulse of the diaphragm. When, on the other hand, the tongue is used to control the airflow, the player can dose pressure more finely. While a weak tongue attack causes a stream of air only slightly over the air pressure threshold to start the sound, a strong diaphragm impulse probably overshoots this threshold, and thus shortens the *rise time*.

The *rise time* of the loud sound with weak tongue attack is relatively short when seen in the context of the other sounds of Table 2: we expected this sound's *rise time* to be over 0.100 s, but several sounds with these characteristics were compared and they all had a *rise time* between 0.085 and 0.090 s. Potentially, for a loud sound with weak attack, the combination of air pressure and strong impulse of the diaphragm has a larger effect on its *rise time* than the weak tongue attack.

For the synchronization task in the experiment, a reference sound was needed. We chose a snare drum click. This click has a very short *rise time* of 0.003 s, so its *PAT* is supposedly nearly identical with its *PhOT* (Rasch, 1988). Other studies used artificial clicks, for example with 2 ms duration (Camp et al., 2011) or spectrally matched clicks (Wright, 2008) as a reference sounds. In our pre-tests, very short clicks turned out to be irritating (which supports Wright's findings) and masking or fusion problems were observed using spectrally matched clicks (contrary to Wright's findings). We finally chose the snare drum rim click as reference sound, because it has a fast rise time, and the drums/sax combination is frequently heard in popular music, hence the click is ecologically valid.

### Participants

A total of 40 participants was recruited, 33 male and 7 female. Their mean age was 28 years (*sd* = 9.9) at the time of the study. All were either professional musicians or enrolled in a BA or MA in Music program with substantial experience on their main instrument (mean = 17 years, *sd* = 9.8). Twenty participants played the saxophone as their main instrument.

### Setup and procedure

Two different methods of determining relative timing between sounds have been applied in previous studies: forced choice temporal order judgment or synchronization judgment. In temporal order judgment experiments, participants hear two sounds and answer which one starts earlier. In synchronization judgment experiments, participants hear two sounds and indicate if they perceive them as being synchronized or not. We used a synchronization judgment approach, which is well established for measuring *PAT* (Villing, 2010).

Past research used three different synchronization judgment task methods for determining *PAT*. In the first approach, participants synchronized an experimental stimulus with a reference sound (Gordon, 1987; Wright, 2008; Camp et al., 2011; Polfreman, 2013). This is based on the assumption that the *PATs* of the two sounds fall together when the two sounds are at the Point of Subjective Simultaneity. In the second method, participants tapped in synchrony with the audio instead of using a reference sound (Vos et al., 1995; Scott, 1998). In the third approach, participants were asked to create an isochronous sequence by shifting an experimental stimulus between fixed reference sounds (Marcus, 1981; Gordon, 1987; Rasch, 1988; Barbosa et al., 2005; Collins, 2006). Villing (2010) compared the methods on a theoretical level, but did not specify which method was overall preferable. In our study, we used the first approach (synchronization of experimental and reference sound), because, in pre-tests, participants found this task to be less challenging, and performed it more quickly than the other tasks. This method creates two acoustical problems: temporal masking (the onset of one sound conceals the onset of the other) and fusion (the two sounds blend into one and become indiscernible). In order to avoid masking (as reported by Gordon, 1987; Villing, 2010; Camp et al., 2011), a clear stereophonic separation between the saxophone sound (left channel) and the snare drum click (right channel) was used. The volume ratio between the two sounds was adjusted by ear to be realistic and comfortable, and was kept constant throughout the study. Fusion is unlikely to occur with this combination of a fast decaying percussive sound like the snare drum click with a sustained gradually induced sound like the saxophone sounds (Gordon, 1987).

The experiment was conducted using a mobile setup with an Apple MacBook Pro (2013 model), a detached Apple Magic Keyboard and Bose AE2 headphones. Each participant was tested individually. For the experiment, participants were seated at a desk in a quiet environment like an empty classroom or library room. The perceptual task was carried out using *Cubase Elements* (version 7.0.80). The experimenter operated the computer; participants were unable to see the screen and used only the detached keyboard to give feedback. Participants were informed about the study and gave written consent.

The participants' experimental task was to move the saxophone sound to a point in time when they heard it perfectly synchronized with the click, sounding like two instruments played perfectly together. Participants changed the location of the saxophone sound using the left and right arrow keys on the keyboard. Each keystroke moved the saxophone sound by 0.001 s either in negative (earlier) or positive (later) direction relative to the snare click. The two sounds were played in a loop, and it was possible to move the saxophone sound while the loop was being played. Participants could stop and restart the playback at any time by pressing the space bar. They notified the researcher when they heard the attack of the saxophone sound and the click simultaneously. The researcher noted the difference between the saxophone note's *PhOT* and the snare drum click's *PhOT* in a spread sheet.

Participants familiarized themselves with the experimental task by synchronizing a piano sound with the snare click. While synchronizing the test example, participants could adjust the overall volume of the headphones to a comfortable level.

Stimuli were presented in a randomized order. For each of the nine trials, there were two standard *initial saxophone sound placements*: either the saxophone sound was initially placed ~0.2 s after the click or ~0.2 s ahead of it. Presentation was counterbalanced, so for one participant the experiment started with four trials in early sax sound *placement* condition followed by five trials in late saxophone *placement* condition. For the next participant, the *initial saxophone sound placements* were flipped: four behind trials, followed by five ahead trials.

After performing the synchronization task, participants answered a number of personal questions about their age and sex, the musical instrument they play, and how long they have been playing it. The time to finish the experiment varied between 20 and 40 min, including short breaks of 1 or 2 min if participants asked for a break.

## Results

### Overview

Analysis of the collected data was conducted in RStudio (version 1.1.423) with R (version 3.3.2). Figures were created with the ggplot2 package (version 2.2.1). The experiment yielded 360 (9 stimuli × 40 participants) valid *PAT* estimates. According to the rules of thumb by Bulmer (1979), the distribution of the *PAT* estimates is approximately symmetric (skewness = 0.487) and slightly leptokurtic (excessive kurtosis = 1.007). Mean *PAT* for the nine stimuli ranged from 0.023 s (soft/strong tongue attack) to 0.083 s (soft/without tongue attack) after *PhOT* with a mean *SD* of 0.027 s per stimuli.

### Onset rise time

**Figure 3:**
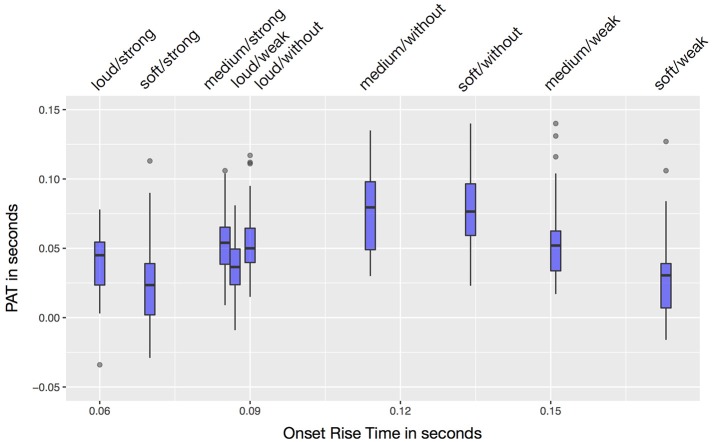
Onset rise time and *PAT* of the nine sounds.

As suggested by earlier research, *onset rise time* was positively correlated with the 360 *PAT* estimates. Yet, this correlation was weak [*r* = 0.143, *t*_(358)_ = 2.748, *p* = 0.006]. Figure 3 shows the *onset rise times* and measured *PATs* for all nine sounds.

### Dynamics and articulation

A two-factor analysis of variance was carried out to measure the effects of *dynamics* and *articulation* on *PAT* (Table 3). Mauchly's test for sphericity was significant for the *articulation* × *dynamics* interaction (*W* = 0.465, *p* > 0.001). Hence, Greenhouse-Geisser correction was applied to the significance probabilities.

**Table 3:** Effects of the playing techniques (articulation, dynamics) on the PAT.

**Effect**	***Df***	**Sum Sq**	**Mean Sq**	***F***	***p***	**η*^2^***
Articulation	2	0.08396	0.04198	55.511	<0.001	0.316
Dynamics	2	0.02603	0.01302	17.213	<0.001	0.098
Articulation × Dynamics	4	0.02502	0.00626	8.272	<0.001	0.094
Residuals	351	0.26544	0.00076			

According to Cohen (1988), and Miles and Shevlin (2000), effect sizes between η^2^ = 0.060 and η^2^ = 0.140 represent a medium effect, while greater effect sizes are considered large. Following these recommendations, the main effect of *articulation* was large (η^2^ = 0.316). The main effect of *dynamics* (η^2^ = 0.098) and the *articulation* × *dynamics* interaction (η^2^ = 0.094) were medium-sized.

**Figure 4:**
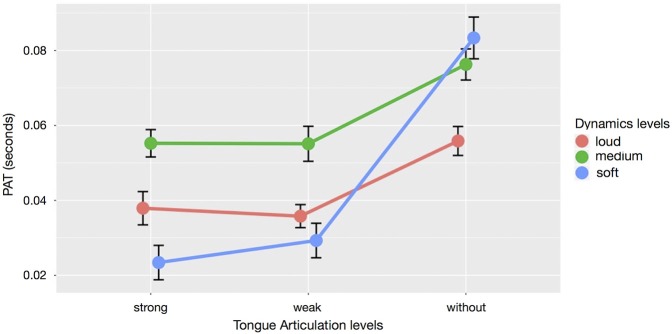
Interaction plot of mean *dynamics* and *articulation* levels with the respective *PAT*s. Error bars indicate standard error of the mean.

The three effects can be studied in the interaction plot of Figure 4. It shows that the effect of *dynamics* on the *PAT* depends on *articulation*. A *post-hoc* Tukey HSD test (see Table 4) revealed that sounds played without tongue attack had a significantly later *PAT* than sounds played with either weak or strong tongue attack (*p* < 0.001). The strength (weak vs. strong) of the attack had no significant influence on PAT (*p* = 0.938). Loud sounds had a significantly earlier *PAT* than medium sounds (*p* < 0.001). In comparison, soft sounds reacted more strongly to changes of *articulation*: the PAT is early for soft sounds with strong or weak tongue attack, but late when the soft sound is articulated without tongue.

**Table 4:** *p*-values for the pairwise comparisons of a Tukey HSD test PAT ~ articulation × dynamics with significant values highlighted.

	**Loud strong**	**Loud weak**	**Loud without**	**Medium strong**	**Medium weak**	**Medium without**	**Soft strong**	**Soft weak**
Loud/weak	1.000							
Loud/without	0.088	**0.033**						
Medium/strong	0.114	**0.044**	1.000					
Medium/weak	0.120	**0.047**	1.000	1.000				
Medium/without	<**0.001**	<**0.001**	**0.027**	<**0.001**	**0.018**			
Soft/strong	0.311	0.536	<**0.001**	**0.020**	<**0.001**	<**0.001**		
Soft/weak	0.896	0.980	<**0.001**	<**0.001**	<**0.001**	<**0.001**	0.989	
Soft/without	<**0.001**	<**0.001**	<**0.001**	<**0.001**	<**0.001**	0.966	<**0.001**	<**0.001**

### Initial saxophone sound placement

The analysis revealed an influence on the *PAT* that was not related to the stimuli themselves: the *initial saxophone sound placement* for the trials. As stated above, the experiment was conducted in two *initial placements*: either the physical onset of the saxophone sound was placed ~0.2 s earlier than the snare drum click at the beginning of the task or the physical onset of the saxophone sound was placed ~0.2 s after the click. The initial placement had a significant effect on the PAT [*t*_(357)_ = 5.269, *p* < 0.001]. The mean *PAT* was earlier for trials with the saxophone sound starting behind the click (*PAT*_behind_ = 0.041 s, *sd* = 0.032) compared to the trials where the saxophone sound was initially placed ahead of the click (*PAT*_ahead_ = 0.059 s, *sd* = 0.032). A Brown-Forsythe type test showed that the variance of the *PATs* was the same for both *initial placements* [*F*_(179)_ = 1.042, *p* = 0.782], hence participants did not perform their task worse in either of the two *initial placements*. A three-way analysis of variance was calculated to measure the effects of *articulation* × *dynamics* × *initial placement* on *PAT*. Since none of the interactions between *initial placement* and the two other predictors was significant, we concluded that *initial placement* does not influence the main and interaction effects of *articulation* and *dynamics* on the *PAT*.

Predicting *PAT* from the *rise time* separately for each *initial placement* did not substantially strengthen the correlation between *PAT* and *rise time* [behind *t*_(178)_ = 2.351, *p* = 0.019, *r* = 0.173; ahead *t*_(178)_ = 1.496, *p* = 0.136, *r* = 0.111]. Fisher's *z* transform was used to compare the two correlation coefficients. The difference between the two correlation coefficients was not significant (*z* = 0.600, *p* = 0.550).

## Discussion

### Playing techniques and rise time

The playing techniques (*articulation* and *dynamics*), had a major influence on the *PAT* of the used saxophone sounds. The main effect of *articulation* was approximately three times larger than the main effect of *dynamics*. There is also a significant *interaction* between the two, having an effect nearly as large as *dynamics*.

The interpretation of the large main effect of *articulation* on the *PAT* location is straightforward: if the sound is played with the tongue, its *PAT* is perceived to occur earlier than when the tongue is not involved as an active articulator. We had hypothesized that strongly articulated sounds would have an earlier *PAT* than weakly articulated sounds. This, however, was not the case: the decisive influence on the *PAT* was the categorical difference whether the tongue was involved in the articulation of a note, not the strength of the tongue's impulse.

The main effect of *dynamics* was that loud sounds have a significantly earlier perceived attack than medium loud ones. This supports Gordon's (1987) findings that louder sounds have an earlier *PAT*. The result further agrees with the finding of Vos and Rasch (1981) that listeners generally hear the *POT* of sounds early if a sound has a high intensity; Boenke et al. (2009) found a similar effect of intensity in their temporal order judgment experiments. For soft sounds in comparison to medium loud and loud sounds, however, we measured a considerable *interaction* effect between *articulation* and *dynamics*: soft sounds were much more affected by *articulation* than medium or loud sounds. One possible explanation for this effect is that the tongue attack might be better audible in the context of a soft sound compared to a louder sound. It is unclear whether differences in presentation level intensity (how loud the sound is played on an audio device, as observed by Boenke et al. (2009)) and production level intensity (e.g., how loud a sound was played on the instrument) lead to the same results.

This study shows that playing techniques like *articulation* and *dynamics* are relevant for the PAT of saxophone sounds and affect their “rhythmic placement” (Wright, 2008). This result potentially applies to other instrumental sounds with complex onsets and envelopes, and may be used in future modeling efforts. In his comparison of models, Villing (2010) showed that the overall best performing model (Pompino-Marschall, 1989) performed poorly when it was applied to complex sounds. Similarly, according to Collins (2006), some models worked well with sine tones but less so with more complex sounds. For instruments like the saxophone, a *PAT* model could be developed using several sounds of the same instrument with different playing techniques or characteristics. This model can then be expanded to accommodate other saxophones and subsequently other reed instruments.

The results support the claim made by Vos and Rasch (1981) and Camp et al. (2011) that analyzing performed rhythm on the basis of inter-onset intervals between the *PhOT* may not be ideal, at least when it comes to instruments with complex onsets: since the distance between *PATs* and *PhOTs* depends on the characteristics of the sounds, the inter-onset intervals between *PhOTs* might represent our perception of the rhythm unreliably.

Some models predict *PAT* from the *rise time* (Vos and Rasch, 1981; Gordon, 1987). The correlation between *PAT* and *onset rise time* was weak in this study (contrasting with findings by Hirsh, 1959; Camp et al., 2011; Barnett-Cowan et al., 2012). The discrepancies might partly be explained with the different *rise times* used in the experimental stimuli across studies: Hirsh's *rise times* varied from 0.002 to 0.015 s, Barnett-Cowan et al.'s were between 0.005 and 0.700 s, whereas in this study, *rise times* varied between 0.060 and 0.173 s. Our findings support Villing (2010) that Gordon's best performing model (which involves *rise time*) may not work well with sounds that have a complex onset (such as speech or wind instruments). However, since the *rise time* was not of main interest in our experiment, it was not systematically varied.

After completing the experiment, most participants stated, that they had never thought about *PAT* and its implications before, even though all of them qualified as professional musicians. As musicians, they have developed a natural feel for playing in synchrony with colleagues over the years, but they do not seem to have a conscious notion of the note onset as a potentially complex event. This natural feel for synchrony seems to have a parallel in speech: in a study by Barbosa et al. (2005), participants were capable of speaking syllables in synchrony to a metronome without any training, and without further knowledge of the concept of *p-centers*.

### Initial saxophone sound placement

In this study, the *initial saxophone sound placement* in the trials had a significant impact on the *PAT* localization, but did not interact with the effects of the playing techniques. Our data does not provide any evidence that one of the two *initial placements* leads to results closer to the actual *PAT*. During the experiment, we observed a tendency in participants to overshoot in either direction. In the initial phase of each trial, participants first displaced the saxophone sound from its early or late position (depending on the *initial placement*) toward the click. When the saxophone sound reached a time interval in which participants did not perceive a better or worse synchronization when moving the sax sound, they had a tendency to push it in the same direction until they heard a difference again, and moved it slightly back to place the *PAT*. Friberg and Sundberg (1995) reported just noticeable time differences of 0.006 s for synchronization tasks. However, the observed mean difference of 0.018 s between mean PATs in the ahead and behind initial placement conditions exceeds the just noticeable difference by far. So, perceptual acuity might account only for a portion of the wide gap between *PATs*.

One potential explanation is that the click masked the saxophone sound onset in spite of the stereo separation. Masking has been reported in experiments involving a synchronization judgment task (Gordon, 1987; Villing, 2010; Camp et al., 2011) and it may be may be a problem inherent to this kind of experimental design. The snare click takes ~0.020 s to decay, during which many participants may not be able to discriminate the timing differences. Temporal masking (Fastl, 1976, 1977) between click and saxophone sound might also be a factor here. A shorter click could have been used to avoid this phenomenon. But it would have been perceived as an artificial sound and it might have triggered other difficulties for the synchronization task (Wright, 2008). Short audio signals have been reported to mask time intervals longer than they last themselves (Fastl, 1976).

### PAT values and density functions

**Figure 5:**
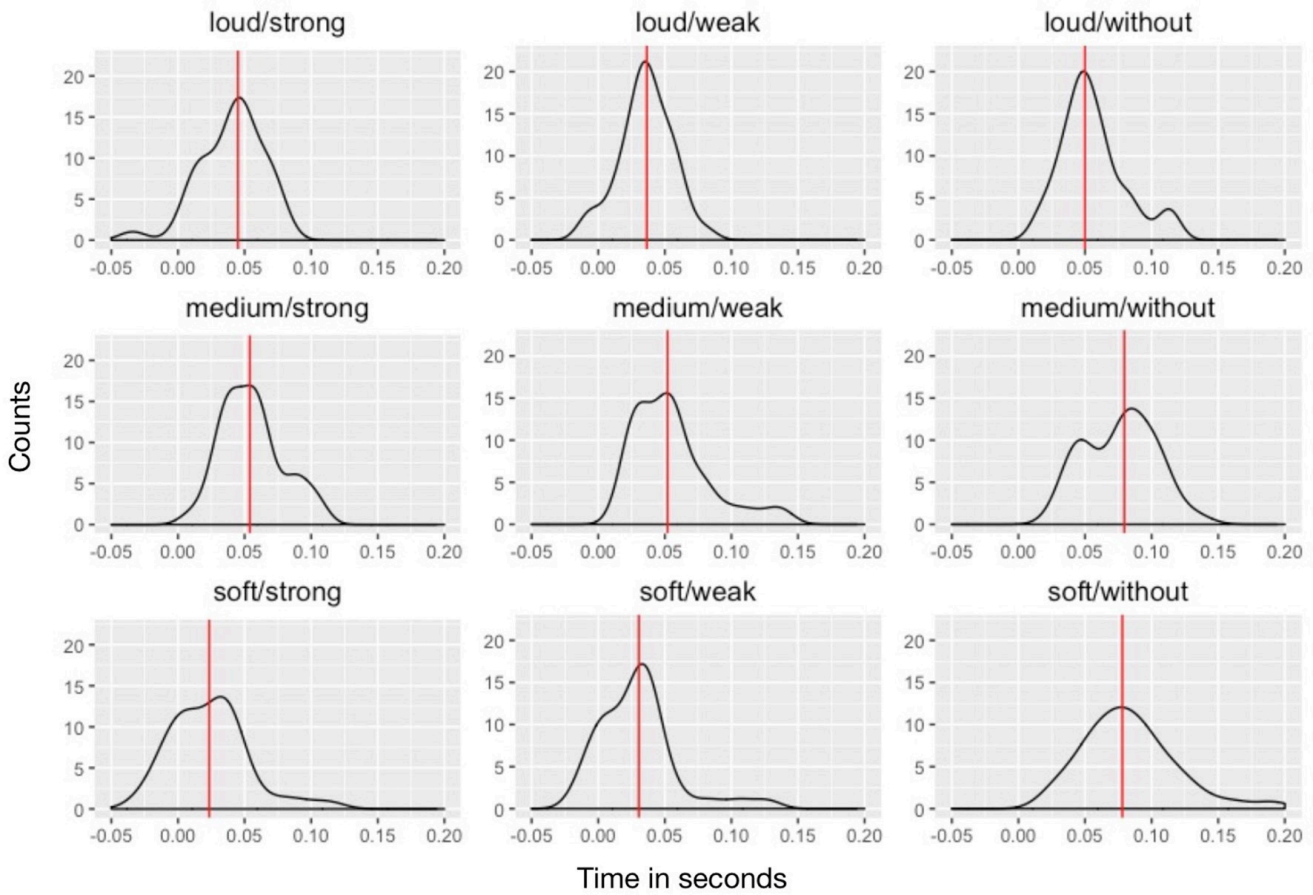
*PAT-pdf* density plots for the nine stimuli. The x-axis is time in s; the y-axis represents *PAT* density. Red lines indicate *PAT* medians.

Gordon (1987) considered *PAT* point estimates to be vague or even meaningless, particularly for stimuli with a long *rise time*. Instead, he proposed describing the *PAT* as a probability density function (*PAT-pdf*), which estimates the probability for each time interval to contain the *PAT*. Wright (2008) and Polfreman (2013) further elaborated on this concept. Figure 5 shows *PAT-pdfs* for each of this study's nine stimuli, based on the experimental data.

This study's *PAT* measurements show considerable spread: standard deviations for the observations on one stimulus ranged between 0.020 and 0.035 s, which agrees with earlier research. We expected that *PAT*-*pdfs* of stimuli with a more percussive onset (e.g., loud dynamics/strong attack) would show less variance than the measurements with smoother onsets, but his was not the case.

According to the *PAT-pdf* concept, the *PAT* is understood as a random variable that takes listeners' variance of PAT perception explicitly into account. However, the usability of the *PAT-pdf* in performance analysis practice is questionable: firstly, the idea that the *PAT* is a random variable is implicit in the whole endeavor of measuring *PAT* experimentally. Secondly, in order to use the *PAT* as a basic tool for analyzing microtiming in performance research, a point estimate is of much greater use than a *pdf* (or confidence intervals based on the *PAT-pdf*). In consequence, we propose to report *PAT* point estimates in perceptual studies, and use them with caution in the analysis of performance. When applying the *PAT* in analysis, we should be aware of the uncertainty connected to the estimate, and refrain from interpreting very small *PAT* or inter-onset interval differences that are not likely to be perceived by a majority of listeners.

## Conclusions

This study investigated the *PAT* of saxophone sounds. We found that the *articulation* and the *dynamics* of the sounds have a significant effect on the *PAT*. The influence of articulation on the *PAT* is quite straightforward: sounds with tongue articulation (weak or strong) have an earlier *PAT* than sounds without tongue articulation. The influence of *dynamics* on *PAT* is less clear: generally, loud and soft sounds had an earlier PAT than the medium loud sounds. Hence we did not find a monotonous (either increasing or decreasing) relationship between *dynamics* and *PAT* location. The interaction between *articulation* and *dynamics* indicates that, in sounds with soft *dynamics*, the placement of the *PAT* is particularly sensitive to the absence or presence of tongue articulation. Together, *articulation, dynamics*, and their interaction explained considerably more *PAT* variance (η^2^ = 0.508) than the *onset rise time* (*R*^2^ = 0.020), an acoustic feature of the sound that was used to model *PAT* placement in past research.

These findings suggest that estimating the *PAT* of saxophone sounds on the basis of *articulation* and *dynamics* is likely to be more accurate than modeling *PAT* with the *rise time* as single predictor. We hypothesize that this sensitivity of the *PAT* to different playing techniques is not unique to the saxophone, but it might be relevant to many other kinds of instruments or voices that manifest complex onset behavior.

Besides the *articulation* and *dynamics* tested in this study, other acoustic features (e.g., pitch), different kinds of articulation (e.g., ghost notes, staccato) or aspects of the performance (e.g., the embouchure and air pressure a player produces, the build and the materials of the instrument) might prove to be relevant for the *PAT* of saxophone sounds. When the focus shifts from examining isolated sound events to compound sound objects like melodies, the context of a sound (e.g., legato playing or the preceding melodic interval, see Almeida et al., 2009 for note transitions on the flute) is potentially relevant.

An entirely different approach for determining *PAT* could be used in future investigations that emphasizes the production side of synchronizing sound events: we might instruct saxophone players to synchronize notes with a metronome or a snare drum click. Players are subsequently asked to identify the notes they think were perfectly synchronized with the click, which then are used to estimate the *PAT*. This method eliminates the influence of the *initial saxophone sound placement*, and masking problems can also be considered to be negligible. To synchronize sounds with a metronome or notes from another player is a familiar task for each musician. This method shifts the focus from how sounds are perceived by listeners to how they are perceived while being played by the musician.

As of today, the project of creating comprehensive models to estimate the *PAT* of saxophone or other wind instrument sounds is still a work in progress. And even if an adequate model can be found, applying it to recorded real-world musical performances in order to analyze microtiming will be a challenge of its own. In performances that involve several musical voices played simultaneously, the identification of any kind of sound features (*PhOT, p*max) is usually problematic. Yet, to recognize any such features in recorded music appears to be a necessary precondition that a model can be applied at all. Further acoustic features that are specific to the sound of interest (e.g., spectral flux or high frequency content) might then prove helpful to anchor the model and its estimates in the substrate of the sound signal.

## Data availability

Datasets and stimuli of this study are included in the Supplementary Files.

## Ethics statement

The Swiss Federal Law on Research on Humans (Humanforschungsgesetz, HFG, from September 30, 2011) specifies that health-related studies must obtain approval by the regional Ethics Commissions (HFG, Art. 45). Our study is not a health study as defined by the law (HFG, Art. 2) and does hence not require to be approved by the regional Ethics Commission. All subjects gave written informed consent in accordance with the Declaration of Helsinki.

## Author contributions

TB and OS conceived the experiment. TB recorded and selected the stimuli. TB recruited the participants. TB analyzed the data. TB and OS wrote the paper.

### Conflict of interest statement

The authors declare that the research was conducted in the absence of any commercial or financial relationships that could be construed as a potential conflict of interest.
